# The Ovulation Theory of Menstruation

**Published:** 1876-12

**Authors:** 


The Ovulation Theory of Menstruation: Will it Stand? By A Reeves Jackson, A.M., M.D., Surgeon-in-chief of the Woman's Hospital of the State of Illinois, etc., etc. Reprint from the American Journal of Obstetrics, October, 1876. New York: Wm. Wood & Co.

The author, in this pamphlet of thirty-four pages gives, we think, fairly the arguments pro and con on this subject, and finds nothing which satisfies him that ovulation, or even the existence of the ovaries, are necessary to menstruation. He has tabulated twenty seven cases in which the ovaries had been removed, in nearly half of which menstruation continued regularly, and in over half the molimen and irregular discharges occurred. He also reports three cases of "normal ovariotomy" as furnished him by Dr. Ely McClellan, of Louisville, Ky., in which menstruation continued regularly as before spaying.

